# The controversial role of nutraceuticals vs. drugs in proliferative retinopathies

**DOI:** 10.3389/fnins.2025.1727089

**Published:** 2026-01-15

**Authors:** Dario Rusciano, Paola Bagnoli

**Affiliations:** 1 Independent Researcher, Catania, Italy; 2 Department of Biology, University of Pisa, Pisa, Italy

**Keywords:** age-related macular degeneration, anti-VEGF therapy, curcuma, diabetic macula edema, diabetic retinopathy, enhancement of antioxidant defenses, interference with angiogenic signaling, Lutein

## Abstract

Neovascular eye diseases, notably age-related macular degeneration and diabetic retinopathy, remain major causes of vision loss despite advances in pharmacological management. The proliferation of abnormal retinal blood vessels leads to the loss of retinal cells and progressive visual dysfunction. Anti-VEGF therapies have revolutionized treatment; however, their efficacy is incomplete, they require repeated administration, and resistance or suboptimal responses are not uncommon. These limitations have stimulated interest in additional therapeutic approaches, both inspired by preclinical research and aimed at improving the management of systemic conditions that contribute to neovascular pathologies. Beyond conventional pharmacology, nutraceuticals have attracted attention for their proposed mechanisms—enhancement of antioxidant defenses, modulation of inflammatory cascades, and potential interference with angiogenic signaling—which provide a molecular rationale for their application in ocular disease. This review critically examines the dual landscape of current pharmacological strategies and nutraceutical approaches, analyzing how the latter might enhance retinal resilience and vascular stability in the early stages of disease. The novelty of this work lies in juxtaposing the mechanistic underpinnings of nutraceuticals with the clinical shortcomings of anti-VEGF therapy, thereby identifying opportunities for integrative therapeutic perspectives. Nevertheless, nutraceuticals cannot replace pharmacological treatment in advanced disease; rather, they may offer incremental benefits in early-stage or high-risk patients, contingent upon timely preventive diagnosis. Until more robust clinical evidence and regulatory oversight are established, nutraceuticals should be regarded as adjunctive components within personalized care models—supporting, but not substituting for, established pharmacological interventions.

## Introduction

Ocular diseases associated with vessel proliferation such as neovascular age-related macular degeneration (nAMD), proliferative diabetic retinopathy (PDR) and its related complication diabetic macula edema (DME), rank among the top global causes of irreversible vision loss among the elderly (Al-Namaeh, 2022; Yuan et al., 2024). In combination, they represent a major and growing public health burden, affecting hundreds of millions worldwide and leading to millions of cases of blindness and severe visual impairment. Despite extensive preclinical and clinical research, the precise molecular mechanisms underlying these pathologies remain incompletely understood, often limiting the available therapies to improve the health disorder without attempting to cure the underlying condition. A recent editorial, highlights that anti-VEGF therapies “can slow the progression but do not cure the disease,” even though they have become the cornerstone of treatment for neovascular age-related retinal diseases (Gozzo et al., 2023). More broadly, available treatments across a range of retinal degenerations remain compensatory rather than curative, targeting symptoms rather than root causes (Khan et al., 2025).

In clinical ophthalmology, patient management often involves identifying individual risk factors to predict disease progression and implementing targeted interventions accordingly. Pharmacological treatments remain central, particularly in conditions like AMD, where numerous prognostic indicators guide therapy (Hagag et al., 2024). Nutraceuticals are also increasingly used as adjuncts, especially in early-stage disease although early AMD is difficult to be diagnosed. Antioxidant formulations such as lutein and zeaxanthin have been shown to slow geographic atrophy progression (Keenan et al., 2024), while curcuma-based nutraceuticals are linked to reduced AMD risk (Alsoudi et al., 2024). In DR, specific dietary strategies may help modulate disease mechanisms (D'Angelo et al., 2025). Clinical summaries also support these integrative approaches, while highlighting their limitations (National Center for Complementary Integrative Health, 2024).

Pharmacological agents such as anti-VEGF therapies are firmly established as first-line treatments for nAMD and DR, supported by extensive clinical trial evidence and strong guideline recommendations (Parravano et al., 2025; Bahr and Bakri, 2023). However, their long-term use is limited by high cost, potential systemic and ocular side effects, and the need for frequent intravitreal injections (Banerjee et al., 2025; Ham et al., 2023). In response, novel drug delivery systems—including biodegradable implants, injectable hydrogels, and nanoparticle-based carriers—are being actively explored to reduce treatment burden while maintaining efficacy (Ham et al., 2023; Luaces-Rodríguez et al., 2020). In parallel, nutraceuticals—bioactive compounds from dietary sources—are gaining attention as preventive or adjunctive strategies, particularly in early-stage nAMD. Formulations containing lutein, zeaxanthin, omega-3 fatty acids, and antioxidants have shown favorable outcomes in delaying disease progression, as demonstrated in recent meta-analyses and clinical studies (Csader et al., 2022; Wang D. et al., 2024; Wang et al., 2024b; Parmar et al., 2025). Nonetheless, their preventive utility remains constrained by the typically late diagnosis of neovascular retinal diseases.

Despite substantial preclinical promise, the real-world efficacy of nutraceuticals in human diseases remains contentious due to variability in study design, poorly characterized participant populations, bioavailability challenges, and inconsistent regulatory oversight. Clinical trials often enroll heterogenous cohorts and rely on complex botanical mixtures whose bioactive components may interact unpredictably (Csader et al., 2022; Satyam and Bairy, 2022). Moreover, compounds such as curcumin, omega-3 fatty acids, and carotenoids generally exhibit low oral bioavailability because of poor solubility, rapid metabolism, and degradation, complicating consistent dosing and therapeutic impact (Nicolescu et al., 2023; Colletti et al., 2022). Regulatory inconsistency across regions further undermines product standardization and quality, making it difficult to distinguish credible outcomes from ineffective formulations (Komala et al., 2023). The possibility of restricting nutraceutical use to a truly preventive context is limited by practical barriers to early detection of retinal diseases—patients are typically diagnosed only once neovascular stages produce noticeable symptoms.

The debate over the roles of nutraceuticals vs. pharmaceuticals in ocular diseases hinges on differences in prevention vs. treatment, cost-effectiveness, and mechanistic clarity (Silpi, 2025), which is further complicated by inconsistencies in regulatory oversight and traditional usage models. Conventional pharmacological treatments excel in acute and advanced clinical scenarios, while nutraceuticals may offer safer, more sustainable options for risk reduction and long-term adjuvant care—though at present, their clinical rigor and standardized evidence base remain less definitive. Above all, the use of nutraceuticals in prevention is limited by the difficulty in achieving widespread early detection of retinal neovascular diseases, which only become treatable when vision damage becomes clearly detectable. This review examines and contrasts both strategies, focusing on molecular mechanisms, clinical outcomes, and how they can best be integrated into ophthalmic practice.

## Neovascular retinal diseases: epidemiology, impact, and the need for integrated management

nAMD and PDR represent vision-threatening endpoints of retinal diseases characterized by pathological angiogenesis. Recent epidemiological studies demonstrate that nAMD affects approximately 2–3% of elderly populations in high-income countries, with incidence rates increasing exponentially with age-rising 1.2-fold in adults aged 75–84 and 2.4-fold in those 85–96, as documented in Finnish population studies between 2006 and 2020 (Korva-Gurung et al., 2023). Globally, the prevalence of any AMD exceeds 10% in individuals over 80 years, with projections suggesting continued growth as populations age (Li et al., 2020; Fleckenstein et al., 2024). DR shows similarly striking progression patterns, affecting nearly 80% of patients after 20 years of diabetes duration, though only about 7% develop the proliferative form that carries the highest risk of vision loss (Solomon et al., 2017; Teo et al., 2021). Therapeutic strategies for these conditions have primarily focused on anti-VEGF agents, which show partial mitigation of oxidative stress markers. However, the persistent progression in some cases highlights the need for therapies addressing upstream pathological drivers, particularly in aging and diabetic populations where fundamental quality control mechanisms decline (Kowluru, 2019). The economic burden extends beyond direct healthcare costs to include lost productivity ($16.3 billion) and increased caregiving needs ($5.5 billion), with total annual costs for AMD estimated at $30.4 billion in the United States (Rein et al., 2022). This impact is particularly severe in low-resource settings where access to advanced treatments is limited, highlighting global disparities in vision health (Yuan et al., 2024).

Anti-VEGF agents have revolutionized the management of nAMD and PDR, significantly improving or stabilizing visual outcomes for many patients (Solomon et al., 2019; Aldokhail et al., 2024). However, these therapies typically require frequent and indefinite intravitreal injections, imposing a substantial treatment burden, and up to 30% of patients exhibit suboptimal responses or progressive vision loss despite therapy (Enríquez-Fuentes et al., 2024). In addition, anti-VEGF treatments address distinct downstream targets of the molecular pathway leading to retinal vessel proliferation while additional targets are ignored although being heavily involved in the angiogenic cascade. Laser photocoagulation, while still employed in PDR management, is primarily preventive—limiting the risk of severe complications—but does not restore lost vision and may result in peripheral field defects (Aldokhail et al., 2024). The persistence of visual decline and disease recurrence in a subset of patients underscores the urgent need for more comprehensive, mechanism-driven strategies that intervene earlier and target multiple levels of disease pathogenesis (Enríquez-Fuentes et al., 2024; Creuzot-Garcher et al., 2022).

A critical gap in current management is the lack of emphasis on prevention and early intervention. The role of systemic disease control in preventing maculopathies is exemplified by conditions such as diabetes (diabetic maculopathy) and hypertension (age-related maculopathy). Additional modifiable risk factors, including smoking, poor dietary habits, and uncontrolled systemic disorders, significantly influence disease onset and progression (Tian and Zhang, 2025; Saigal et al., 2025; Hassan et al., 2024). In nAMD, smoking approximately doubles the risk of disease development, whereas in PDR, glycemic control remains the most important modifiable determinant (Kuan et al., 2021; Khunti et al., 2025). As confirmed in a recent comprehensive review, foundational evidence from the DCCT/EDIC study established that early, intensive glycemic control reduces the long-term risk of PDR by 52%—a finding that continues to underscore the critical window for preventive intervention (Tecce et al., 2024; Nathan, 2014). Moreover, population studies indicate that adherence to a Mediterranean-style diet is associated with a significantly lower risk of AMD (Keenan et al., 2020).

The future of neovascular retinal disease management requires a paradigm shift that extends beyond purely pharmacological interventions. While anti-VEGF therapies and laser treatments remain indispensable, their limited capacity to modify the underlying disease process and their high treatment burden highlight the need for complementary approaches that address upstream pathological mechanisms—particularly oxidative stress, chronic inflammation, and metabolic dysregulation. Emerging, mostly preclinical, evidence suggests that nutraceuticals such as omega-3 fatty acids, carotenoids, and polyphenols may influence these pathways, potentially enhancing the efficacy of conventional therapies or delaying disease progression in high-risk individuals (D'Angelo et al., 2025; Blasiak et al., 2019).

However, critical gaps persist. Current nutraceutical research lacks standardized formulations, robust clinical endpoints, and long-term safety data, leaving their role in adjuvant care unresolved (Puri et al., 2022). To translate preclinical promise into clinical practice, future efforts must prioritize:

- Mechanistic synergy: Conducting rigorous clinical trials to evaluate nutraceutical–pharmacological combinations (e.g., anti-VEGF with antioxidants) and determine whether additive or synergistic benefits exist. Recent work on co-delivery systems combining antioxidant compounds with VEGF-targeted agents highlights both the translational potential and the methodological challenges of such strategies (Dias et al., 2025). Supporting this concept, a randomized clinical trial demonstrated that omega-3 supplementation given concomitantly with intravitreal anti-VEGF therapy significantly reduced vitreal VEGF levels in patients with neovascular AMD (Rezende et al., 2014). Nonetheless, limitations persist, including the complexity of defining measurable clinical endpoints and the confounding influence of dietary variability.
- Biomarker-driven prevention: Identifying high-risk phenotypes (e.g., genetic predispositions or metabolic profiles) that may benefit most from early dietary or nutraceutical intervention.
- Accessible integration: Developing cost-effective, evidence-based protocols for adjunctive nutraceutical use—particularly in underserved populations where adherence is challenging—must reckon with regulatory complexity and gaps in clinical validation. Reviews of regulatory frameworks highlight fragmented oversight and weak transparency in nutraceutical approvals (Komala et al., 2023), while methodological analyses argue that conventional clinical trial designs often fail to accommodate nutraceutical-specific constraints (Evans et al., 2022).

Therefore, while standalone nutraceuticals are unlikely to replace pharmacological therapies, their potential as complementary tools warrant cautious optimism. A collaborative approach—uniting ophthalmologists, nutrition scientists, and public health experts—is essential to validate their efficacy, optimize delivery, and redefine standards of care for neovascular retinal diseases. Nevertheless, a major limitation remains the current inability to achieve early diagnosis, which constrains the timely application of nutraceutical strategies. Market pressure for billion-dollar revenues, driven by consumer demand for perceived health benefits, is counterbalanced by scientific and regulatory challenges, fostering skepticism within the scientific community due to the lack of consistent evidence and persistent safety concerns.

## The impact of late diagnosis and pharmacotherapy limitation

Neovascular retinal diseases progress silently until advanced stages, when structural damage limits treatment efficacy and recovery potential.

Over the past two decades, therapeutic advances—most notably anti-VEGF agents—have transformed management paradigms. Agents such as ranibizumab, aflibercept, and brolucizumab are widely used to suppress pathological angiogenesis and reduce macular edema, with the goal of stabilizing or improving visual acuity. However, the effectiveness of these treatments is highly dependent on early detection and prompt initiation. Clinical and real-world evidence consistently demonstrate that patients diagnosed and treated in the early stages of disease achieve significantly better visual outcomes and long-term stability compared with those receiving delayed therapy (Teo et al., 2025; Borrelli et al., 2025).

Yet, the effectiveness of anti-VEGF therapy in the real world is limited by significant rates of non-persistence and non-adherence, with meta-analyses estimating that approximately one-third of patients do not continue their prescribed treatment course (Shahzad et al., 2023). Moreover, these therapies are not universally effective even among treated patients. A large-scale analysis revealed that over 4 years, only 59% of nAMD patients maintained stable vision, while nearly 15% experienced severe vision loss despite therapy (Ciulla et al., 2022). This treatment limitation is further exacerbated over the very long term, with studies showing over 25% of patients can have very poor visual acuity after a decade of treatment (Cheema et al., 2021). Similarly, in PDR, while pan-retinal photocoagulation and anti-VEGF therapies significantly reduce the risk of severe complications, a recent meta-analysis confirms they do not restore damaged retinal architecture and can contribute to adverse effects like peripheral vision loss or aggravated macular edema (Macaron et al., 2025). Furthermore, response to anti-VEGF in nAMD is influenced by systemic comorbidities, as the presence of diabetic retinopathy is a significant independent risk factor for poorer long-term visual outcomes (Boscia et al., 2024).

Beyond their biological limitations, current pharmacological therapies also impose a substantial economic burden. Anti-VEGF treatments require frequent administration—typically monthly or bimonthly—particularly in the initial years of therapy. The average cost per intravitreal injection ranges from $1,500 to $2,000 USD, depending on the agent and healthcare system. Most patients receive 6 to 12 injections annually, leading to treatment costs of $25,000–$50,000 in the first two years and often exceeding $100,000 over a decade when ancillary imaging, monitoring, and travel are included (Rein et al., 2022). In advanced cases of PDR, the financial burden is further compounded by surgical interventions such as vitrectomy, which carry not only higher costs but also increased risks and variable outcomes depending on systemic comorbidities and the severity of retinal damage.

**Figure 1:**
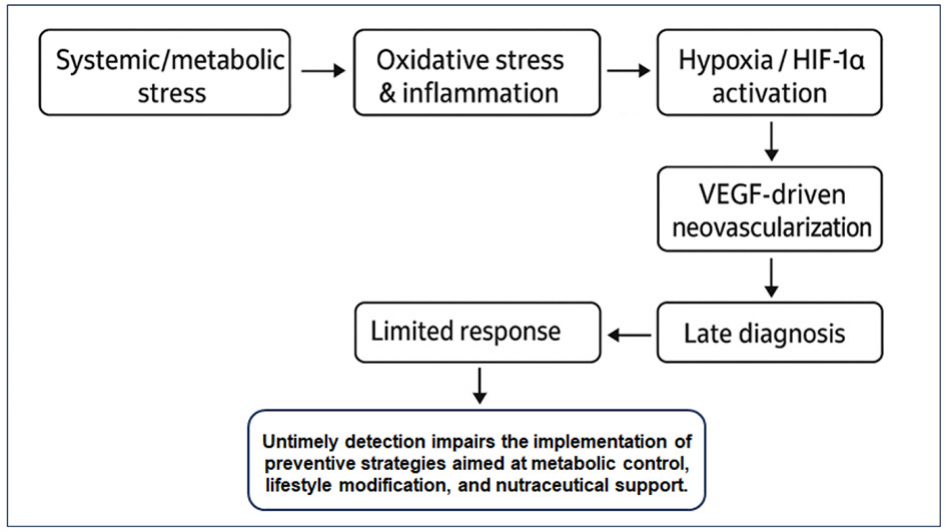
Pathophysiological timeline linking oxidative stress, inflammation and late diagnosis to treatment outcomes.

In real-world settings, many patients present only after significant vision loss has occurred due to poor disease awareness or delayed access to care, narrowing the therapeutic window and limiting recovery, since the retina lacks intrinsic regenerative capacity (Figure 1). Consequently, late-stage interventions remain largely reactive, aimed at halting further deterioration rather than restoring vision or addressing upstream disease mechanisms.

As this gap becomes more evident, the discussion is shifting toward earlier detection and integrated care models. While the consequences of delayed diagnosis and the cost-effectiveness of current pharmacological options are well understood, earlier-stage interventions—including regular eye examinations, lifestyle modifications, and dietary supplementation—are increasingly recognized for their potential to modify disease progression. However, despite the growing demand for retinal imaging, the limited number of ophthalmologists and trained eye technicians remains a major obstacle to the early diagnosis of ocular diseases. At the same time, the debate concerning the limitations of anti-VEGF therapy and the need for additional therapeutic strategies is somewhat constrained by the reality that it remains the only currently available treatment capable of directly addressing retinal neovascularization. This should not be viewed as resignation, since ongoing research continues to refine anti-VEGF agents and delivery systems while exploring complementary approaches—such as nutraceuticals—within a more rigorous scientific and regulatory framework.

## Molecular mechanisms underlying neovascular diseases of the eye

The molecular mechanisms underlying neovascular pathologies of the eye are extremely complex and cannot be described as a simple cascade of events, but are better defined as a network of interactions containing several critical nodes, each representing a potential therapeutic target. However, any molecular cascade triggered by ischemic insult to the retina ultimately converges on the upregulation of vascular endothelial growth factor (VEGF), the major effector in the proangiogenic process (Li and Huang, 2025). Retinal ischemia results from reduced blood flow and consequent oxygen and nutrient deprivation; aging, lifestyle factors, hypertension, diabetes, and genetic predisposition are among its main causes. The ensuing hypoxia initiates a cascade of cellular events leading not only to retinal cell death and potential vision loss but also to compensatory vessel proliferation. These fragile, newly formed vessels—particularly in neovascular AMD—tend to leak blood and fluid, promoting hemorrhage and fibrotic scarring that may culminate in retinal detachment and severe visual impairment.

### Hypoxia coupling to HIF-1

From ischemia to neovascular diseases, a starting point is the activation of Hypoxia-Inducible Factor 1 (HIF-1), a transcription factor that plays a crucial role in cellular adaptation to oxygen deficiency. Once activated by low oxygen tension, HIF-1 regulates the expression of genes involved in various processes, including neovessel formation to compensate for reduced oxygen availability (Magar et al., 2024). In normal oxygen conditions, HIF-1α is continuously produced but rapidly degraded by the proteasome via prolyl hydroxylases (PHDs), enzymes that require oxygen. Reduced oxygen levels prevent HIF-1α degradation, thus leading to accumulation of HIF-1α. HIF-1α dimerizes with HIF-1β and the heterodimer translocates to the nucleus where it binds to hypoxia-response elements (HREs) in target gene promoters. Once bound, HIF-1 activates transcription of genes involved in various cellular responses to hypoxia. Among the transactivated genes is VEGF, which stimulates the formation of new blood vessels to improve oxygen supply. HIF-1 activity is not only regulated by oxygen levels but also by intracellular pathways such as PI3K/Akt (see below). As a further layer of regulation, HIF-1 can induce transcription of its own regulators (e.g., PHDs), thereby promoting HIF-1α degradation and helping to attenuate the hypoxia response (Magar et al., 2024).

### G-protein coupled receptor (GPCR) coupling to HIF-1

GPCR-mediated signaling contributes to the adaptive cell responses to low oxygen tension through an intricate network of transduction pathways converging on HIF-1. As a paramount example in the retina, hypoxia induces overactivation of the sympathetic system that leads to an increased release of norepinephrine, which links to its beta-adrenergic receptors (β-ARs) located at different retinal levels. Of them, β2-ARs localized to Muller cells couple dually to G-proteins either stimulatory (Gs) or inhibitory (Gi) both converging on adenylyl cyclase (AC). In the Gs pathway, increased AC activity results in elevated cAMP that activates protein kinase A (PKA), which, in turn, phosphorylates a number of intracellular proteins that converge on HIF-1 that transactivates a number of proangiogenic genes including VEGF (Cammalleri et al., 2024). The final HIF-1-associated player VEGF activates its receptor VEGFR2 to then reverberate on the PKA pathway either directly or through the PI3K/Akt signaling to ultimately converge on HIF-1. Sympathetic overstimulation activates the β2-AR-Gi signaling pathway that counteracts the Gs-mediated generation of cAMP. Gi-associated alternative signaling cascade includes the PI3-K/Akt pathway that regulates HIF-1 expression while the extracellular signal-regulated kinase 1/2 (ERK1/2) signaling mediates accumulation of reactive oxygen species (ROS) by endothelial nitric oxide synthase (eNOS) uncoupling, a major cause of endothelial dysfunction (Villadangos and Serrador, 2024). In respect to the proangiogenic activity of β2-AR, the last cloned receptor in the β-AR family, there are indication that β3-AR acts by protecting the retina from neovessel proliferation through a compensation of VEGF upregulation (Cammalleri et al., 2023). This is in line with the increasing success of β3-AR agonism to treat overactive bladder (Peyronnet et al., 2025) and its expanding translational role in the diseased heart (Michel et al., 2025) and obesity (Samanta et al., 2024). In this respect, preclinical studies demonstrated that β3-AR agonism may counteract the effects of hyperoxia in the ileum of newborn rats thus extrapolating its use to neutralize the damaging effects of sudden exposure to hyperoxia induced by preterm birth (Nardini et al., 2024, 2025).

### HIF-1 coupling to reactive oxygen species (ROS) and inflammation

Both ROS and inflammation play a crucial role in the progression of neovascular diseases of the eye. During hypoxia, ROS are produced by mitochondria as a byproduct of their normal function in oxidative phosphorylation. They result from electrons leaking from the electron transport chain and interacting with oxygen. Increased production of ROS, leads to the inhibition of PHD activity and subsequent stabilization of HIF-1α protein that allows HIF-1α to accumulate in the cytoplasm. This would occur through the production of hydrogen peroxide that oxidizes ferrous iron (Fe^2+^) to its ferric form (Fe^3+^) thus preventing the binding of ferrous iron to PHD and limiting HIF1α hydroxylation that allows HIF-1α to accumulate in the cytoplasm. Excess ROS trigger inflammation, which in turn amplifies oxidative stress, creating a self-perpetuating cycle. Both ROS and inflammatory signals interact with HIF-1α in a complex and reciprocal manner with both inducing HIF-1α expression and stabilizing its protein level, while HIF-1α that affects ROS production and inflammation. In particular, ROS stimulates the expression of NF-κB (a nuclear factor kappa-light-chain-enhancer of activated B cells), a protein complex that functions as a transcription factor, which plays a crucial role in regulating gene expression related to inflammation. ROS-induced inflammatory signals including cytokines, directly impact upon HIF-1 signaling through the regulation of HIF-1 at transcriptional and post-translational levels finally affecting HIF1α protein accumulation (Malkov et al., 2021; Michalak and Michalak, 2025). Conversely, HIF-1α signaling regulates the release of inflammatory cytokines by activating downstream signaling pathways coupled to the generation of inflammatory components.

### From HIF-1 to VEGF

**Figure 2:**
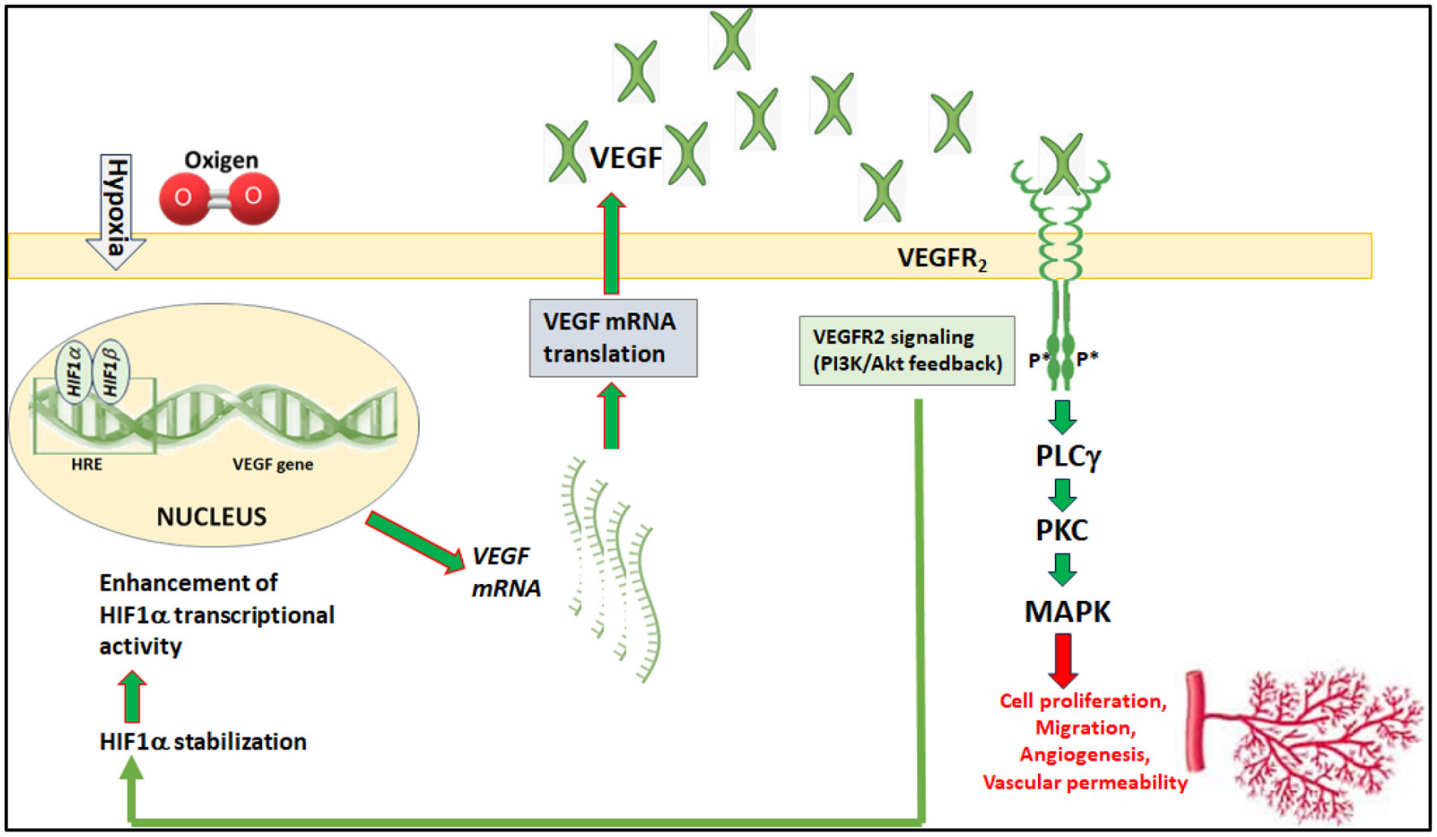
VEGF regulation of neovessel proliferation. Hypoxia enhances HIF-1α transcriptional activity leading to increased expression of VEGF, which binds to VEGFR2. Its activation by the ligand drives endothelial cell proliferation through the MAPK pathway, finally leading to pathological neovascularization. VEGFR2 signaling reverberates on HIF-1α stabilization through the PI3K/Akt pathway thus participating to HIF-1α accumulation. Abbreviations: HRE, hypoxia-responsive element; PLC, phospholipase C; PKC, protein kinase C; MAPK, mitogen-activated protein kinase; PI3K/AKT, phosphatidylinositol 3-kinase/Akt kinase also called PKB or protein kinase B.

Once transactivated by HIF-1α binding to HRE, the VEGF gene undergoes transcription into mRNA that is then processed to the VEGF protein, which then performs its functions related to angiogenesis and cell survival. In a simplistic view, VEGF acts by binding to its receptors on endothelial cells to then trigger a cascade of events that promote endothelial cell proliferation, migration, and survival, ultimately leading to the development of new blood vessels. This process is essential for both normal physiological functions like embryonic development and wound healing, as well as for pathological conditions including neovascular eye diseases. VEGF binds to its receptors that are a family of transmembrane receptor tyrosine kinases of which VEGFR2 binding to VEGF leads to the phosphorylation of the tyrosine residues and activation of the kinase domain. VEGFR2 phosphorylation activates signaling pathways leading to neovessel proliferation (Figure 2). The damaging effect of proliferating vessels is additionally complicated by the increased vascular permeability at the blood retinal barrier (BRB) level that heavily impacts on visual function. Among the factors influencing BRB leakage, the endocytosis of cadherin, a key endothelial cell adhesion molecule, is associated to VEGFR2 phosphorylation (Smith et al., 2020). Therefore, inhibiting the VEGF-VEGFR2 signaling can help to reduce the growth of abnormal malfunctioning blood vessels by preventing the activation of VEGFR2 signaling. Several drugs targeting VEGFR-2 have been approved and are currently utilized to halt the pathological axis of VEGF-VEGFR (Shah et al., 2025; Wang et al., 2024a). Moreover, recent work in fundus neovascularization summarizes in detail the downstream signaling mechanisms and therapeutic strategies aimed at the VEGF/VEGFR2 axis in the retinal environment (Li and Huang, 2025).

### Vascular health and retinal cell integrity

Proper blood vessel function is essential for retinal health, supplying cells with oxygen and nutrients. When perfusion falls, oxygen deprivation rapidly triggers retinal cell loss. On the other hand, when retinal vessels proliferate to compensate for low oxygen tension, ischemic conditions in the central avascular zone additionally leads to hypoxia and subsequent cell death.

In the widely used oxygen-induced retinopathy (OIR) model, which mimics retinopathy of prematurity (ROP) in preterm infants, different retinal cell types undergo various forms of programmed cell death triggered by oxidative stress, inflammatory mediators released by activated microglia, and dysregulated signaling pathways. In particular, ischemic retinopathy affects not only vascular endothelial cells but also astrocytes, retinal neurons, Müller cells, and other glial elements (Nian et al., 2021). Over the OIR phases, hyperoxia can directly cause neuronal and endothelial cell death, while the subsequent hypoxic phase contributes to cell loss through polyamine oxidation and dysregulated autophagy, whose inhibition may represent a therapeutic strategy in OIR. In particular, an excess of ROS disrupts the balance in endogenous antioxidant systems, resulting in oxidative stress and consequently induces retinal cell death (Zhou et al., 2025). A range of endogenous protectants—neurotrophic factors, antioxidant enzymes such as superoxide dismutase and catalase, and mechanisms like autophagy—preserve retinal integrity by limiting ROS-induced damage and supporting neuronal survival, representing key targets for therapeutic intervention (Zhou et al., 2025; Zhu et al., 2025; Liu et al., 2010; Gao et al., 2025; Xin et al., 2022; Osborne et al., 2018). Superoxide anion is scavenged by superoxide dismutase to form H_2_O_2_ and O_2_. High levels of H_2_O_2_ can generate the hydroxyl radical, which is highly reactive and harmful to nucleic acids, proteins, and lipids in the presence of iron. This is particularly noteworthy in preterm infants with low plasma transferrin and a reduced ability to bind excess iron (Ruan et al., 2024). Glutathione plays a key role as an antioxidant as it rapidly detoxifies H_2_O_2_, thus preventing its reaction with iron and the formation of the hydroxyl radical. It is also a key regulator of lipid peroxides and its inactivation often results in accumulation of lipid peroxides and death. Among endogenous antioxidants, the Pigment epithelium-derived factor (PEDF) prevents photoreceptor apoptosis in the OIR model by inhibiting oxidative stress and endoplasmic reticulum stress. PEDF reduces ROS production and protects cellular structures like mitochondria from damage, thereby acting as a neuroprotective agent against ischemic damage in the retina (Wang Y. et al., 2024). Additional neuroprotectants include members of the VEGF family that may play a role as possible modulators of the strictly interconnected communication between the vascular and neuronal compartments in the retina. Members of the VEGF family that are traditionally known to play a role in vessel formation, are increasingly recognized for their neuroprotective role that extends beyond vascular effects (Aksan and Mauceri, 2025). In particular, a tight coupling between RGCs and capillaries has been described possibly through molecular mechanisms involving VEGF although mechanisms underlying the VEGF neuroprotection remain elusive (Froger et al., 2020).

Despite intensive efforts to optimize anti-VEGF therapy for cancer and retinal disease, it is important to consider potential long-term consequences of sustained VEGF inhibition, since VEGF also exerts trophic and homeostatic effects in neural and vascular tissues. VEGF-A and related family members have well-documented neuroprotective actions in the retina—promoting retinal ganglion cell survival, Müller-cell viability, and production of neurotrophic factors via VEGFR2 → PI3K–Akt signaling—so prolonged suppression could, in principle, reduce endogenous neurotrophic support (Banerjee et al., 2025; Nishijima et al., 2007; Foxton et al., 2013). Clinically, several lines of evidence raise concern about structural and functional sequelae after long courses of intravitreal anti-VEGF. Multiple observational and meta-analytic studies have reported an association between prolonged anti-VEGF therapy and the development or progression of macular atrophy/geographic atrophy in eyes treated for nAMD, although disentangling the effects of the underlying disease from therapy remains challenging (Vofo et al., 2025; Eshtiaghi et al., 2021). Real-world, long-term series also show that mean visual acuity gains achieved in the first years after treatment tend to decline over longer follow-up, suggesting either incomplete rescue of retinal function or late degenerative changes not prevented by VEGF blockade (Corazza et al., 2021; Spooner et al., 2025). Other documented safety signals include agent-specific risks (for example, higher rates of intraocular inflammation reported with some molecules), and pharmacovigilance analyses emphasize variability in ocular adverse-event reporting across anti-VEGF agents (Lakhani et al., 2025). In special populations, such as preterm infants treated for ROP, concerns have been raised about possible effects on retinal vascular maturation and systemic development after intravitreal anti-VEGF exposure, underscoring the need for careful long-term follow-up (Sukgen et al., 2022). Finally, although systemic exposure from intravitreal dosing is low, reviews of systemic safety note isolated reports and signals—particularly in patients with preexisting comorbidities—prompting continued surveillance of possible cerebrovascular, renal, or other systemic effects (Banerjee et al., 2025).

Taken together, these mechanistic and clinical observations do not argue against the clear, sight-saving benefits of anti-VEGF therapy in neovascular disease. Rather, they call for (1) vigilance in long-term monitoring, (2) research into treatment strategies that minimize unnecessary chronic VEGF suppression (for example, individualized dosing intervals, alternative targets, or combination approaches), and (3) trials that specifically examine neuroprotective outcomes and structural endpoints (e.g., macular atrophy, choriocapillaris integrity, retinal nerve-fiber health) in addition to traditional fluid- and vision-based measures (Foxton et al., 2013; Eshtiaghi et al., 2021).

## Alternative pharmacological treatments to anti-VEGF therapy

Large-scale analyses of over 130,000 eyes confirm that anti-VEGF therapy is highly effective in routine clinical practice for preserving vision and reducing disease activity across nAMD and DME, over the longer term (Ciulla et al., 2022). The durability of this approach is underscored by studies focusing on specific agents, such as real-world data demonstrating that bevacizumab as a first-line therapy can maintain stable visual acuity in nAMD patients over a 5-year period, highlighting its sustained effectiveness and role in treatment strategies (Mhmud et al., 2025). The therapeutic arsenal has since expanded to include brolucizumab and faricimab, with the phase 3 TENAYA and LUCERNE trials demonstrating that faricimab with a treat-and-extend protocol delivers strong visual and anatomical outcomes at 2 years, offering sustained disease control with fewer injections (Khanani et al., 2024). Furthermore, innovation continues with sustained-delivery systems, as shown by the Pagoda trial, where continuous ranibizumab via a port delivery system was effective for treating DME, representing a significant advancement in treatment modality (Khanani et al., 2025). This evolution from foundational trials to modern real-world validation and innovative delivery systems underscores the sustained success of VEGF inhibition, which remains the cornerstone of management for these vision-threatening conditions.

Yet real-world clinical outcomes consistently fall short of the efficacy benchmarks set by rigorous clinical trials (Shahzad et al., 2023). This gap is driven by a combination of suboptimal treatment adherence and inherent limitations in therapeutic response. A large-scale analysis of real-world outcomes revealed that over a 4-year period, a significant proportion of patients experience suboptimal outcomes, with nearly 15% suffering severe vision loss (a decline of ≥30 letters) despite receiving therapy (Ciulla et al., 2022). The mechanisms underlying suboptimal response are multifactorial. Tachyphylaxis—a diminishing therapeutic effect over time—is a well-documented phenomenon and may involve receptor desensitization, immune activation, or compensatory angiogenic signaling (Sharma et al., 2023). Furthermore, treatment resistance is frequently linked to specific patient comorbidities. For instance, the presence of DR has been identified as a significant independent risk factor for poorer long-term visual outcomes in patients receiving treatment for nAMD, highlighting the role of systemic vascular health in treatment efficacy (Boscia et al., 2024). Other factors such as persistent intraocular inflammation, uncontrolled diabetes, or extensive retinal ischemia also contribute to a diminished treatment response.

In addition to variable efficacy, the treatment burden of anti-VEGF therapy remains high. The standard regimen, established in pivotal clinical trials, requires intravitreal injections on a monthly or bimonthly basis in the first year of therapy (Rosenfeld et al., 2006; Heier et al., 2012). This intensive regimen contributes significantly to undertreatment, poor patient adherence, and attrition from care (Rowe and Ciulla, 2024; Reitan et al., 2023). The consequence of this burden is evident in a recent meta-analysis of real-world use, which demonstrates that visual acuity gains achieved in year one decline progressively over the long term, with greater injection frequency being crucial for sustained outcomes (Spooner et al., 2025). This finding is reinforced by clinical commentaries, which emphasize that undertreatment in real-world practice, compared to the intensive regimens of randomized trials, results in suboptimal visual outcomes for patients (Chakravarthy et al., 2022). Attempts to mitigate injection frequency through innovations such as port delivery systems (e.g., ranibizumab) and dual-action molecules like faricimab offer partial relief by extending treatment intervals (Khanani et al., 2025; Yufeng et al., 2025). However, these advances primarily address treatment burden and do not overcome the fundamental pathophysiological limitation of targeting a single signaling axis in a complex, multifactorial disease (Cabral et al., 2017). Indeed, VEGF is only one element in a broader pathological cascade. Ischemic damage in the retina activates an intricate web of proangiogenic, inflammatory, and oxidative stress-related signals. In the HIF system, HIF-1α primarily upregulates the expression of genes associated with angiogenesis of which VEGF is the leading actor joined by additional genes including among others platelet-derived growth factor, fibroblast growth factor, and stromal-derived factor-1. Meanwhile, chronic inflammation perpetuates endothelial dysfunction through cytokines like interleukin-6, tumor necrosis factor-alpha and monocyte chemoattractant protein-1 that represent attractive therapeutic targets. Additionally, intervening on the expression of neurotrophic factors at different time points might be useful to counteract retinal cell damage despite their several limitations including their hydrophilic nature that prevents them to cross the blood-retinal barrier, their short half-lives and poor pharmacokinetics. While the search for novel pharmacological targets continues to expand the therapeutic arsenal for neovascular retinal diseases, growing interest is also being directed toward non-pharmaceutical approaches—particularly nutraceuticals—which may offer complementary benefits by targeting upstream disease mechanisms such as oxidative stress and inflammation.

Given these complexities, relying solely on VEGF blockade is increasingly seen as inadequate. Many research efforts are thus now directed toward discovering new pharmacological targets, testing complementary drug classes, or integrating adjunctive strategies such as nutraceuticals or gene regulation tools. These alternatives aim to reduce the frequency of invasive procedures, improve efficacy in non-responders, and intervene earlier in the disease process.

In search for additional therapies, major effort is focused in identifying novel targets for developing further medications that can escape the limitations of the anti-VEGF therapy. In addition, nutraceuticals are put on the market despite the skepticism of the medical community.

## Novel targets for additional pharmacotherapy

Expanding the pharmacologic arsenal for neovascular retinal diseases requires going beyond symptomatic control and addressing the core molecular mechanisms of disease. A growing body of research is now centered on the identification of novel molecular targets, with the aim of developing more durable, multi-mechanistic, and potentially earlier-stage interventions. These include regulators of angiogenesis, inflammation, vascular integrity, and retinal cell protection

Upstream transcriptional regulators such as HIF-1α and nuclear factor kappa B (NF-κB) are critical initiators of neovascular and inflammatory signaling. HIF-1α regulates genes like VEGF, PDGF, and FGF in response to hypoxia (Ramakrishnan et al., 2014) while NF-κB controls a wide spectrum of inflammatory mediators including cytokines such as TNF-α and IL-6, chemokines, adhesion molecules, and inflammatory enzymes (Guo et al., 2024).

Drugs interacting with transcription factors may act at multiple levels, either by affecting their binding to DNA or by influencing their post-translational modifications. However, since transcription factors represent points of convergence for multiple signaling pathways and regulate numerous downstream genes, directly targeting them can have broad and unpredictable consequences. Conversely, modulating proteins that regulate transcription factor activity may offer more specific and safer therapeutic opportunities for nAMD and other ocular neovascular diseases. One such example is the inhibition of the redox activity of the recently identified protein reduction-oxidation effector factor 1 (Ref-1) (Muniyandi et al., 2025). Ref-1 regulates several redox-sensitive transcription factors that control key mediators of ocular angiogenesis and inflammation. It acts by reducing disulfide bonds within transcription factors, thereby enhancing their DNA-binding ability and promoting the transcriptional activation of multiple angiogenic and inflammatory mediators. In preclinical models, inhibition of Ref-1′s redox activity has shown growth-inhibitory effects on angiogenesis that in some assays surpass those achieved by anti-VEGF, suggesting it may complement or in certain contexts exceed VEGF-targeted approaches (Hartman et al., 2025).

Acting downstream of transcription factors involves multiple molecular targets, some of which have already advanced to clinical application. For instance, the angiopoietin/Tie (tyrosine kinase with immunoglobulin and epidermal growth factor homology domains) signaling pathway, which plays a central role in angiogenesis, has been successfully targeted by faricimab—a bispecific antibody that simultaneously inhibits VEGF-A and angiopoietin-2 (Ang-2). Large Phase III trials (TENAYA and LUCERNE) have shown that faricimab offers extended durability with visual outcomes comparable to aflibercept, supporting its regulatory approval and integration into clinical practice (Khanani et al., 2024). Similarly, the integrin pathway—involved in endothelial adhesion and migration—has yielded promising clinical candidates. The small-molecule integrin antagonist THR-687 has shown preclinical efficacy in reducing choroidal neovascularization (Hu et al., 2019) and demonstrated tolerability and anatomical benefit in early-phase trials for DME (Khanani et al., 2021). In the context of anti-inflammatory pathways, the complement system, particularly factors such as C3 and C5, is being actively explored in advanced AMD. Agents like pegcetacoplan (APL-2) and avacincaptad pegol (Zimura) have shown clinical efficacy in slowing progression of geographic atrophy in Phase II and III trials, and their potential role in neovascular forms is now under investigation (Qin et al., 2021; Wilke and Apte, 2024).

Beyond inhibiting neovascularization, protective strategies against neovessel-associated retinal cell damage include potential pharmacotherapies targeting neuroprotective signaling pathways within the retina. Among retinal neuropeptides, recent research has underscored the strong protective efficacy of pituitary adenylate cyclase–activating polypeptide (PACAP, primarily the 38–amino-acid isoform) and its receptor PAC1. Jansen et al. (2025) demonstrated that targeted deletion of PAC1 in retinal neurons exacerbates ganglion cell loss, axonal degeneration, and microglial activation in a murine model of optic neuritis, indicating that PAC1 signaling is not only protective during inflammatory insults but also supports homeostatic neuronal survival (Van et al., 2021). Furthermore, PACAP signaling mitigates retinal vascular and neuronal damage in models of oxygen-induced retinopathy, reducing avascular zones and preserving function (Kvarik et al., 2021). Importantly, long-term administration of PACAP in adult mice promotes neural regeneration and modulates microglial activity, suggesting durable protective effects in the mature retina (Denes et al., 2023). While nutraceuticals may exert antioxidant or anti-inflammatory effects that partially overlap with PACAP-mediated mechanisms, these findings emphasize the importance of targeting validated neurotrophic pathways.

## Nutraceuticals and dietary supplements

The rising interest in dietary supplementation for retinal disorders reflects an evolving understanding of disease pathophysiology and a shift toward more sustainable, patient-centered management strategies. In this context, nutraceuticals—a term describing bioactive compounds from dietary sources with potential pharmacological benefits—have gained significant attention. It is important to note that from a regulatory standpoint, these products are legally classified as dietary supplements. The following sections will use the term nutraceuticals to discuss their biological mechanisms and clinical potential, while the specific term dietary supplements will be employed when addressing their formal regulation and legal status.

Pharmacological treatments, while indispensable in halting or reversing acute disease processes, often target the consequences of pathology rather than its origins. In contrast, nutraceuticals aim to influence upstream biological mechanisms and systemic risk factors that contribute to the onset and progression of retinal diseases. This preventive and adjunctive perspective is particularly appealing in the context of chronic conditions such as AMD and DR, where early-stage modulation of oxidative stress and inflammatory pathways may confer long-term benefit (Fanaro et al., 2023).

Unlike prescription drugs, nutraceuticals are non-invasive, widely accessible, and typically well tolerated, making them suitable for long-term use even in asymptomatic stages. Their potential therapeutic role is bolstered by a growing body of preclinical research showing that diverse bioactive compounds—including carotenoids, vitamins, omega-3 polyunsaturated fatty acids, and trace elements—can protect retinal cells and vasculature. In animal and cell models, these compounds have been shown to neutralize reactive oxygen species, stabilize mitochondrial function, and modulate proinflammatory cytokine production (Ye et al., 2024; Kumar and Talwar, 2024). For example, *in vivo* studies combining antioxidants with omega-3 fatty acids reported structural protection against light-induced retinal degeneration (Attallah et al., 2024). More broadly, recent reviews of ocular nutraceuticals emphasize overlapping mechanisms of antioxidation, anti-inflammation, mitochondrial support, and vascular stabilization as foundational to their potential role in retinal disease prevention (Parmar et al., 2025; Delmas et al., 2025).

Among the best-characterized compounds are the macular carotenoids lutein and zeaxanthin, which accumulate in the central retina and function as intrinsic blue-light filters and antioxidants. Their presence has been consistently associated with increased macular pigment density and a reduced risk of phototoxic damage, particularly in aging retinas (Lem et al., 2021). Similarly, long-chain omega-3 fatty acids such as DHA and EPA, abundant in neuronal membranes, preserve retinal integrity and modulate angiogenic and inflammatory pathways relevant to both AMD and DR (Fathima et al., 2023; Weir et al., 2023; Zeppieri et al., 2024). These biological effects align with known cellular stressors implicated in neovascularization and microvascular damage, supporting their role in retinal protection.

Evidence from human studies further supports the potential role of supplementation in modifying disease risk and progression. The Age-Related Eye Disease Studies (AREDS and AREDS2) remain the most influential trials in this context, demonstrating that specific combinations of antioxidants and minerals can reduce the likelihood of progression to advanced AMD in selected patient populations (Age-Related Eye Disease Study Research Group, 2013). However, the magnitude of benefit observed in these trials was moderate, and the effects were confined to patients with intermediate disease at baseline. These findings underscore a broader truth in the field: the clinical impact of supplementation appears to be highly dependent on timing, patient phenotype, and the precise formulation used.

Although some studies suggest additive or synergistic effects when nutraceuticals are used alongside pharmacologic agents (Lafuente et al., 2019), definitive conclusions remain elusive due to the limited number of well-controlled trials designed to test such combinations. Nonetheless, the idea that nutraceuticals could serve not as stand-alone treatments but as integral components of a layered therapeutic strategy is gaining traction. In this model, supplementation may help maintain homeostasis in high-risk individuals, support retinal resilience under metabolic stress, and potentially extend the interval between pharmacological interventions in patients already receiving intravitreal therapies (Semeraro et al., 2019; Datseris et al., 2025).

**Figure 3:**
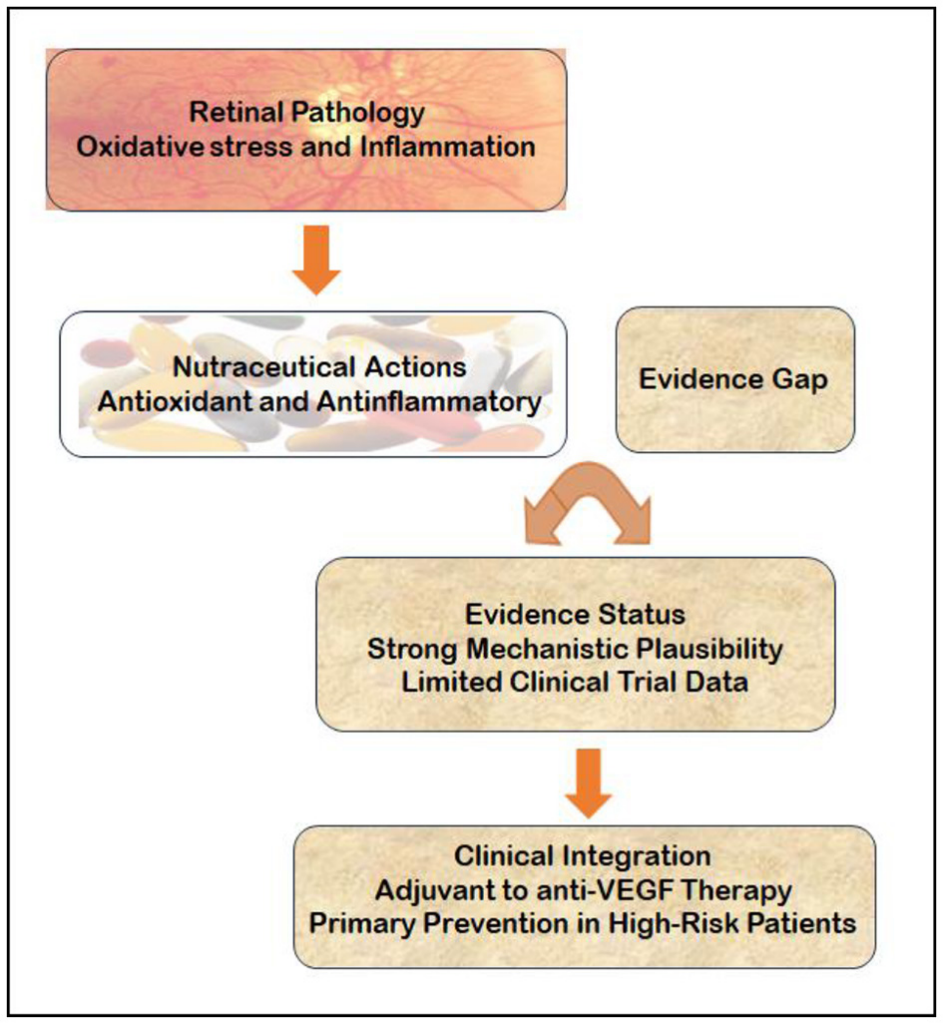
Schematic overview of nutraceuticals in neovascular retinal disease. The figure highlights the rationale for nutraceuticals use (retinal protection through antioxidant and anti-inflammatory actions), the current imbalance between mechanistic plausibility and clinical evidence, and a proposed pathway integrating molecular mechanisms, evidence appraisal, and clinical decision-making.

The use of nutraceuticals in retinal care remains fraught with methodological challenges (Figure 3). Variability in dosing, formulation quality, and study endpoints has rendered many trials difficult to compare and unable to inform standardized clinical guidelines. Difficulties in creating appropriate placebos and maintaining blinding, combined with frequent dropout and poorly defined control groups, further weaken the evidence base. This uncertainty is exacerbated by baseline nutritional heterogeneity among participants and a regulatory landscape ill-suited to supplement research (Yong et al., 2015; Weaver and Miller, 2017).

Despite these limitations, dietary supplementation continues to represent a promising frontier in retinal medicine—particularly as part of a multifactorial approach to disease prevention and progression control. Its integration into clinical care will require greater clarity in formulation standards, improved patient stratification methods, and rigorous outcome-based research. These issues, along with the regulatory context and clinical nuances of specific micronutrients, will be further addressed in the following sections.

### Regulation of dietary supplements

The regulation of dietary supplements represents a critical—and often controversial—aspect of their clinical integration, particularly in fields such as ophthalmology where efficacy, safety, and consistency are paramount. Unlike pharmaceuticals, which are subject to stringent regulatory oversight and rigorous pre-market evaluation, supplements occupy a more loosely governed domain. The rules that define their production, marketing, and surveillance vary not only across countries but also in terms of enforcement and scientific thresholds, resulting in substantial disparities in product quality, consumer protection, and clinical confidence.

In the United States, the foundational legal framework for dietary supplements was established with the passage of the Dietary Supplement Health and Education Act (DSHEA) in 1994. This act formally categorized dietary supplements as a subset of food rather than as drugs, thereby exempting them from the rigorous pre-approval requirements imposed by the Food and Drug Administration (FDA) for pharmaceuticals. Under DSHEA, manufacturers are responsible for ensuring that their products are safe and that their labeling is truthful and not misleading; however, they are not required to demonstrate clinical efficacy or obtain pre-market approval before marketing their products. This regulatory distinction continues to shape the current supplement industry and its oversight structure (Coates et al., 2024).

As a consequence of this regulatory latitude, the U.S. supplement market has expanded dramatically over the past three decades. From an estimated 4,000 products in 1994, the number has grown to more than 85,000 dietary supplement products available in the U.S. - as estimated in 2018 (Bailey, 2020) - reflecting not only heightened consumer interest in wellness and prevention but also a regulatory environment that facilitates market entry. This explosion in availability, however, has not been paralleled by equivalent gains in quality assurance or product standardization. Numerous investigations have uncovered wide variations in ingredient composition, dosage accuracy, and contamination with undeclared substances, raising persistent concerns about product integrity and reproducibility (Geller et al., 2015; Zheng and Navarro, 2015).

Although the FDA retains post-marketing authority to remove unsafe products from the market, its ability to enforce these actions is constrained by limited resources and the burden of proof required to demonstrate harm. This reactive model, rather than a proactive, evidence-based oversight system, has drawn increasing criticism from the scientific community and public health advocates. Proposals to modernize DSHEA—such as requiring mandatory product registration, third-party testing, or stronger enforcement mechanisms—have been discussed, but substantive reforms have yet to be implemented (Coates et al., 2024).

In contrast, the regulatory environment in the European Union offers a more centralized and science-driven approach. The European Food Safety Authority (EFSA), established in 2002, plays a pivotal role in assessing the safety and validity of health claims made for supplements and novel food ingredients. EFSA requires substantial clinical or mechanistic evidence before approving any product claims, and this high evidentiary bar reflects a markedly different approach from the U.S. model. However, because actual enforcement still depends on individual member states, the resulting regulatory landscape remains heterogeneous. This patchwork contributes to confusion over product efficacy and safety, particularly when clinicians or consumers compare health claims made under EU authorization with those marketed elsewhere (Vero and Gasbarrini, 2012; de Boer, 2021). Despite this rigor, enforcement remains decentralized: national regulatory authorities retain discretion in interpreting and implementing EFSA guidance, leading to variability in how supplements are approved, labeled, and monitored in different EU countries.

This fragmentation complicates efforts to harmonize supplement use within clinical practice and introduces uncertainty for healthcare providers attempting to navigate national vs. EU-level guidelines. For clinicians and researchers alike, this can pose significant obstacles when comparing studies or establishing uniform recommendations across borders. Moreover, many countries in Europe allow products to be sold with general health claims that stop short of therapeutic assertions, creating a gray zone between scientifically validated interventions and marketing-driven positioning.

The problem becomes even more pronounced in the context of ocular supplements. Products marketed for preserving vision or slowing the progression of conditions such as age-related macular degeneration (AMD) often carry implied—if not overt—claims of disease prevention or mitigation. While certain formulations, most notably those evaluated in the AREDS and AREDS2 trials, are supported by rigorous clinical evidence, the strength of this evidence is limited by the modest magnitude of the observed benefits. In contrast, the vast majority of vision-related supplements available on the market do not adhere to comparable scientific standards. Their composition and dosage are often inconsistent, with variable quality control that complicates direct comparison and clinical evaluation, particularly in populations that fall outside the narrow inclusion criteria of major clinical trials (Alsoudi et al., 2023).

Products marketed for preserving vision or slowing the progression of conditions such as age-related macular degeneration (AMD) often carry implicit or explicit claims of disease prevention. Although the formulations tested in the AREDS and AREDS2 trials remain the only ones supported by large, randomized clinical studies, their benefits were modest and date back more than a decade. The absence of more recent large-scale confirmatory studies raises legitimate questions about the generalizability and current clinical relevance of those findings. In contrast, the majority of vision-related supplements now available lack comparable clinical validation. Many products contain inconsistent or subtherapeutic dosages, omit key nutrients, or include untested ingredients—factors that complicate objective comparison and clinical decision-making, particularly outside the narrowly defined populations of major trials (Alsoudi et al., 2023; Lawrenson and Grzybowski, 2015). More recent reviews confirm this evidence gap: while preclinical studies continue to demonstrate antioxidant, anti-inflammatory, and mitochondrial-protective effects of natural compounds, large-scale human trials remain scarce, leaving most claims unverified (Parmar et al., 2025; Wang D. et al., 2024; Hu et al., 2024). These inconsistencies raise safety concerns and hinder reproducibility, ultimately undermining both scientific credibility and consumer trust.

Given these challenges, it is essential that the scientific community, regulatory authorities, and healthcare professionals advocate for more rigorous standards in supplement development, marketing, and post-market surveillance. Until such measures are widely adopted, clinicians must navigate this complex landscape with caution—favoring products with validated compositions and demonstrated clinical efficacy, while maintaining a critical stance toward unsubstantiated claims. In ophthalmology, where long-term patient compliance, subtle disease progression, and functional outcome measures all complicate treatment evaluation, this regulatory gap remains a major barrier to the safe and effective integration of supplements into clinical care models. Moreover, dietary clinical trials themselves face inherent limitations, including numerous confounding variables and methodological challenges, which complicate the translation of findings into clinical practice (Mirmiran et al., 2021). Nevertheless, these scientific and regulatory limitations have done little to curb the strong public demand for supplements or the substantial profits generated by the companies that market them.

### Nutraceuticals in neovascular eye diseases

Neovascular diseases of the retina are sustained by pathological angiogenesis, oxidative stress, and chronic inflammation—processes that extend beyond the local ocular microenvironment (Srejovic et al., 2024). While anti-VEGF therapy remains the therapeutic cornerstone, it does not fully halt disease progression in all patients and fails to address the underlying metabolic and inflammatory dysregulation that predispose to vascular pathology (Sharma et al., 2023). Over the past decade, an ever-expanding body of experimental literature has explored the potential of nutraceuticals and dietary bioactives to counter these mechanisms. Hundreds of nutrients, phytochemicals, and functional compounds have been investigated for their antioxidant, anti-inflammatory, neuroprotective, or antiangiogenic effects, generating a vast and heterogeneous evidence base. Given this abundance of data, the present section does not aim to provide an exhaustive review but rather to focus on a selected group of nutraceuticals whose mechanistic relevance, preclinical consistency, and emerging clinical findings render them particularly pertinent to neovascular eye diseases. Accordingly, the following discussion highlights representative compounds—carotenoids (lutein, zeaxanthin, meso-zeaxanthin), omega-3 polyunsaturated fatty acids, polyphenols and flavonoids, as well as vitamins and trace elements—that exemplify the molecular and functional diversity of nutraceuticals with potential roles in modulating retinal angiogenesis, oxidative stress, and vascular stability (Lee et al., 2024).

Micronutrients such as folate and vitamin D have been implicated in the maintenance of vascular homeostasis (Wolf and Kenney, 2019). Folate plays a critical role in the remethylation of homocysteine, and hyper-homocysteinemia is known to promote endothelial dysfunction and vascular permeability. In experimental models of ROP, folic acid supplementation has been associated with improved vascular regulation and reduced neovascularization, suggesting a potential for nutritional modulation of early-stage retinal angiogenesis (Nian et al., 2025).

Similarly, epidemiological research has consistently explored the association between vitamin D status and the development of late-stage AMD. A systematic review and meta-analysis concluded that lower circulating concentrations of 25-hydroxyvitamin D [25(OH)D] were significantly associated with a higher likelihood of advanced forms of AMD, including the neovascular subtype. This inverse association was supported by mechanistic considerations, given vitamin D's documented roles in downregulating inflammatory cytokines and inhibiting angiogenic pathways implicated in choroidal neovascularization (Annweiler et al., 2016). More recently, a large population-based analysis using data from the NHANES 2005–2008 cohort found that individuals with higher serum 25(OH)D levels had a significantly lower risk of developing late-stage AMD, reinforcing the hypothesis that adequate vitamin D status may confer protection against neovascular retinal pathology (Fu et al., 2023). Although observational studies and meta-analyses have revealed a significant association between vitamin D deficiency and DR, several investigations suggest that this relationship diminishes once potential confounding factors such as glycemic control, renal function, and obesity are accounted for. A meta-analysis of 14 observational studies involving over 10,000 participants found that vitamin D deficiency was associated with a higher risk of diabetic retinopathy (pooled OR ≈ 1.27, i.e., an odds ratio indicating a 27% higher likelihood of disease among deficient individuals) and lower serum 25(OH)D levels among affected patients, particularly in proliferative forms (Zhang et al., 2017). Supporting this association in type 2 diabetes, a recent case-control study found vitamin D deficiency to be significantly linked with retinopathy, further emphasizing the potential role of vitamin D status in disease manifestation (Castillo-Otí et al., 2021). However, many original cohort and cross-sectional studies included in that meta-analysis reported that the association became non-significant after statistical adjustment for metabolic and renal variables, indicating that vitamin D status may not independently contribute to disease severity. Furthermore, a systematic review and meta-analysis focusing on sight-threatening DR found that low vitamin D was strongly linked to PDR (OR ≈ 1.8), but the strength of this association was graded as low credibility due to confounding, heterogeneity, and potential bias (Trott et al., 2022). These findings support the view that, although vitamin D deficiency correlates with diabetic retinal disease, its independent role is attenuated in multivariate analyses—highlighting the dominant effect of metabolic control, renal function, and obesity.

Beyond observational data, a robust body of experimental research has elucidated the mechanisms by which nutraceuticals may protect the retina in neovascular diseases. A prominent contribution to this field has come from rodent models of proliferative retinopathies, providing foundational evidence for the protective role of nutritional interventions.

A key study on such models employed a polyethylene glycol (PEG)-induced model of retinal degeneration, which mimics aspects of early dry AMD and shares common inflammatory and angiogenic features with neovascular forms. In this model, the administration of a balanced fatty acid-based dietary supplement significantly counteracted molecular and structural damage induced by PEG-400. The supplement attenuated the expression of key mediators involved in complement activation, angiogenesis (including VEGF), inflammation, and gliosis, and it reduced macrophage infiltration into the choroid. Histological analysis revealed that supplementation preserved the thickness of the outer retina and prevented Müller cell activation, highlighting its capacity to stabilize both neuronal and vascular components of the retina (Cammalleri et al., 2017).

Building on evidence of inflammation-driven retinal damage, a recent study investigated the protective effects of an antioxidant compound in streptozotocin (STZ)-induced diabetic rats, a model sharing features with proliferative retinopathies such as DR and OIR. The treatment—composed of homotaurine, vitamin B6, and other antioxidants—significantly attenuated blood-retinal barrier breakdown and visual dysfunction. Mechanistically, the compound reduced oxidative stress markers (e.g., nitrotyrosine) and suppressed pro-inflammatory mediators, including VEGF, IL-1β, and ICAM-1, while mitigating microglial activation. The therapy also preserved retinal structure, decreasing Müller cell gliosis and preventing neuronal apoptosis, suggesting broad neuroprotective efficacy. These findings align with prior studies demonstrating that targeting oxidative stress and inflammation can ameliorate both vascular and neuronal damage in retinopathies (Canovai et al., 2022).

These findings were synthesized in a recent review, which emphasized the dual antioxidant and anti-inflammatory action of nutraceuticals in proliferative retinopathies (Rusciano and Bagnoli, 2023). The authors argue that these compounds may be particularly effective in the early phases of disease, when oxidative stress and cytokine signaling play pivotal roles in triggering pathological angiogenesis. Importantly, they underscore that such effects are not merely supportive but mechanistically relevant, as nutraceuticals can modulate the expression of transcription factors like NF-κB and HIF-1α, and stabilize the retinal environment under hypoxic and inflammatory stress.

In a broader physiological context, these protective effects must be interpreted within the framework of redox homeostasis. As elaborated in a 2024 review by the same authors, the retina's vulnerability to oxidative stress arises from its high metabolic rate, intense light exposure, and high oxygen consumption. While antioxidants are crucial in mitigating ROS-induced damage, the authors caution that an excessive suppression of oxidative signaling may also be detrimental, potentially disrupting physiological processes such as autophagy and angiogenic balance. Thus, dietary supplementation must aim not for maximal antioxidant activity, but for a physiologically balanced modulation of oxidative stress—a concept particularly relevant to the use of polyunsaturated fatty acids, which both influence membrane dynamics and serve as precursors to anti-inflammatory lipid mediators (Rusciano and Bagnoli, 2024).

These experimental findings are consistent with the broader conceptual framework developed in recent reviews, which propose that the therapeutic potential of nutraceuticals in neovascular eye diseases lies in their ability to support retinal resilience under stress, rather than directly reversing advanced pathology. Table 1 provides a mechanistic overview of major nutraceutical categories, outlining their predominant cellular pathways, reported effects, and the type of evidence supporting their use. In particular, the “Clinical Context & Evidence” column denotes whether findings derive from preclinical models (including cell cultures, rodent models such as OIR and diabetic retinopathy models) or from clinical studies in humans. Given the extremely heterogeneous literature—and the fact that many compounds have been tested across multiple species and experimental paradigms—the table is not intended as an exhaustive model-by-model catalog. Instead, specific experimental models and their outcomes are discussed in detail in Section 7.2. Particular emphasis has been placed on nutraceuticals that act on mitochondrial stability, glutamate excitotoxicity, oxidative burden, and microglial activation—each of which plays a role in the pathogenesis of neovascular and neurodegenerative conditions (Rusciano and Bagnoli, 2023).

**Table 1:** Summary table of nutraceutical targets in neovascular retinal pathologies.

**Nutraceutical category**	**Specific agents**	**Primary molecular & cellular targets**	**Proposed mechanism of action**	**Clinical context & evidence**
Antioxidants & minerals (AREDS-2 formula)	Vitamin C, Vitamin E, Zinc, Copper	Reactive Oxygen Species (ROS), Retinal Pigment Epithelium (RPE)	Scavenges free radicals, reduces oxidative damage to RPE and photoreceptors. Zinc supports antioxidant enzyme function.	Strongest evidence. AREDS-2 formula reduces risk of progression to advanced AMD by ~25% in high-risk individuals (Age-Related Eye Disease Study Research Group, 2013).
Macular carotenoids	Lutein, Zeaxanthin, Meso-Zeaxanthin	Macular Pigment, ROS, Blue Light	Forms macular pigment that filters blue light; acts as a local antioxidant; reduces lipofuscin formation.	Strong evidence. AREDS-2 replaced Beta-Carotene with Lutein/Zeaxanthin. Shown to increase macular pigment density and reduce progression (Lem et al., 2021; Age-Related Eye Disease Study Research Group, 2013).
Omega-3 fatty acids	Docosahexaenoic acid (DHA), Eicosapentaenoic Acid (EPA)	Inflammatory pathways (e.g., NF-κB), Specialized Pro-Resolving Mediators (SPMs: Resolvins, Protectins)	Incorporated into cell membranes; precursors to SPMs that actively resolve inflammation; may downregulate VEGF expression.	Mixed evidence. Some large studies show a modest benefit for reducing AMD progression, others do not. Mechanistically sound for inflammatory and vascular components (Fathima et al., 2023; Weir et al., 2023; Zeppieri et al., 2024; Rezende et al., 2014; Cammalleri et al., 2017; Canovai et al., 2022).
Polyphenols & plant extracts	Curcumin, Resveratrol, Ginkgo Biloba, Bilberry Extract	Nrf2 Pathway, NF-κB, Inflammatory Cytokines, VEGF	Activates Nrf2, boosting endogenous antioxidant defenses; directly inhibits NF-κB to reduce inflammation; mild anti-angiogenic effects.	Preclinical & early clinical. Strong mechanistic data in lab models. Human clinical trial evidence for retinal efficacy is limited and not yet conclusive (Alsoudi et al., 2024; Fanaro et al., 2023; Attallah et al., 2024; Delmas et al., 2025; Canovai et al., 2022).
Mitochondrial supporters	Coenzyme Q10, Acetyl-L-Carnitine	Mitochondrial Electron Transport Chain, ROS	Supports cellular energy (ATP) production in highly metabolic photoreceptors; reduces mitochondrial-derived oxidative stress.	Limited/Emerging Evidence. Primarily theoretical and based on the high energy demand of the retina. Direct evidence for retinal diseases is sparse (Rusciano and Bagnoli, 2023, 2024).

This table summarizes representative nutraceuticals with reported activity against key pathogenic mechanisms underlying neovascular retinal diseases, including oxidative stress, inflammation, mitochondrial dysfunction, and pathological angiogenesis. For each compound or class, the principal molecular targets, experimental or clinical evidence, and putative therapeutic effects are outlined. The selection reflects compounds supported by consistent preclinical findings or emerging clinical data—rather than an exhaustive listing—highlighting the diversity of nutritional approaches explored as adjunctive strategies to conventional anti-VEGF therapy.

Emerging evidence also points toward a systemic dimension in the efficacy of nutritional interventions. A recent pilot study evaluating the adjunctive use of micronutrients in patients receiving intravitreal anti-VEGF therapy for nAMD demonstrated that supplementation was associated with improved visual function and modulation of gut-derived metabolites. These findings raise the possibility of a gut–retina axis, in which dietary components may influence retinal disease via immune-metabolic signaling (Baldi et al., 2024).

Despite these encouraging developments, the clinical translation of dietary supplementation remains hampered by several limitations. Most human studies are observational, of limited size, and often lack standardized formulations or rigorous endpoints. Furthermore, many commercially available nutraceuticals are inadequately tested, vary widely in composition, and are marketed with unverified claims. As has been noted in critical appraisals of the field, this disconnection between scientific rationale and clinical evidence complicates the task of ophthalmologists seeking to provide evidence-based nutritional advice (Lawrenson and Grzybowski, 2015).

Taken together, current evidence indicates that carefully selected nutraceuticals may complement pharmacological therapies within a personalized treatment framework. In high-risk individuals—identified through genetic, inflammatory, or imaging biomarkers—targeted supplementation could enhance therapeutic responsiveness or slow disease progression. While preclinical studies provide strong mechanistic support for these multitargeted compounds, translating such findings into clinical benefit will depend on biomarker-guided approaches, standardized formulations, and robust, well-controlled trials.

**Figure 4:**
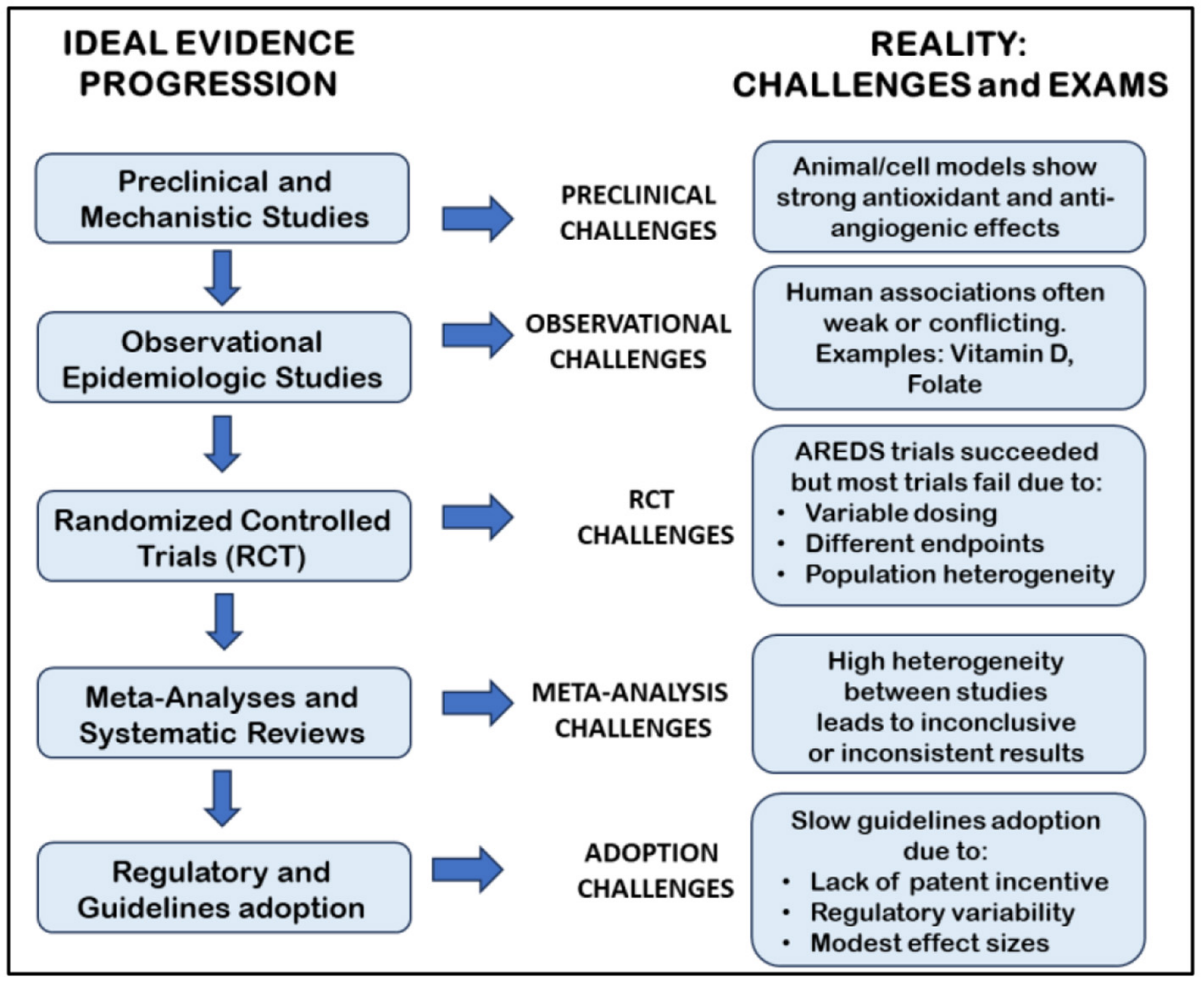
Translational pathway and evidence gaps in nutraceutical research for retinal diseases. The figure summarizes the progressive stages of evidence—from preclinical studies to regulatory adoption—highlighting major limitations that hinder clinical translation. Despite promising antioxidant and antiangiogenic effects in experimental models, most human studies show variable outcomes, high heterogeneity, and slow regulatory uptake due to inconsistent dosing, trial design, and limited standardization.

Within this evolving paradigm, nutraceuticals are best regarded not as curative treatments but as components of a risk-modifying approach to care. Their therapeutic potential may reside in their ability to stabilize vulnerable retinal environments, support metabolic resilience, and complement conventional pharmacologic interventions. As personalized medicine continues to advance, the integration of evidence-based nutritional support may ultimately enhance the long-term management of neovascular eye diseases (Figure 4).

However, nutraceutical demand also reflects the underlying need to correct dietary deficiencies. In populations with balanced eating patterns—such as those adhering to a traditional Mediterranean diet—the overall adequacy of micronutrient intake is generally higher, reducing the necessity for supplemental use. This may in part explain the comparatively lower consumption of nutraceuticals in Mediterranean countries, where diet alone can often provide sufficient levels of essential nutrients (Massimino et al., 2023; Oliver Olid et al., 2024).

## The future of vision care

The management of neovascular retinal diseases is undergoing a transformative shift, driven by convergent advances in artificial intelligence, molecular medicine, and regenerative therapies. The current paradigm, heavily dependent on repeated intravitreal anti-VEGF injections, will likely evolve into a more personalized, durable, and proactive model of care.

A cornerstone of this evolution will be the integration of Artificial Intelligence (AI) and machine learning into clinical workflows. AI algorithms are already demonstrating robust capability in screening and diagnosing retinal diseases from fundus and OCT imaging (Alqahtani et al., 2025; Parmar et al., 2024). The next frontier lies in their application to predict disease progression and therapeutic response. For instance, models are being developed to forecast individual outcomes to anti-VEGF therapy, potentially guiding drug selection and optimizing treatment intervals to maximize efficacy and reduce injection burden (Zhang et al., 2025; Ragni et al., 2024). The future may see AI systems integrating multimodal data—from imaging and genetics to systemic biomarkers—to generate comprehensive prognostic profiles and enable truly personalized treatment protocols.

Concurrently, the therapeutic arsenal is expanding beyond conventional biologics. Gene therapy represents a paradigm shift from chronic treatment to a potential one-time intervention. Strategies using adeno-associated virus (AAV) vectors to enable sustained intraocular production of anti-angiogenic proteins (e.g., anti-VEGF Fab) are showing promise in early clinical trials (Odio-Herrera et al., 2025). The goal of these approaches is to achieve long-term suppression of pathological angiogenesis with a single administration, thereby alleviating the significant treatment burden associated with current regimens (Rowe and Ciulla, 2024). Complementing this, cell-based therapies, particularly the transplantation of stem cell-derived retinal pigment epithelium (RPE), aim to address the underlying cellular dysfunction in diseases like AMD, offering the potential to stabilize and perhaps restore retinal structure (Sakai et al., 2025).

Beyond their established role in diagnosis and prognosis, AI tools may also contribute to overcoming two of the most persistent challenges in the field of nutraceutical research—variable dosing and formulation heterogeneity. By integrating clinical, genetic, and metabolomic datasets, AI-driven models could identify patient-specific nutrient requirements or predict optimal combinations of bioactive compounds to maximize therapeutic synergy while minimizing redundancy or toxicity (Lawrenson and Grzybowski, 2015; Baldi et al., 2024). Similarly, machine-learning algorithms could help standardize evidence across heterogeneous formulations by modeling dose–response relationships derived from real-world data and stratifying subjects according to dietary patterns or microbiome composition (Massimino et al., 2023; Oliver Olid et al., 2024). Such data-centric approaches would move nutraceutical development closer to the precision standards already emerging in pharmacology, facilitating regulatory harmonization and evidence-based personalization (Table 2).

**Table 2:** Comparative overview of major therapeutic strategies in neovascular retinal disease.

**Therapeutic strategy**	**Mechanism/primary target**	**Main advantages**	**Principal limitations**	**Clinical stage/outlook**
Pharmacologic (anti-VEGF, anti-inflammatory, complement inhibitors)	Inhibition of VEGF/VEGFR2, cytokine modulation, complement blockade	Proven efficacy in vision preservation; standardized dosing; well-established safety monitoring	Requires repeated injections; limited effect on upstream drivers (oxidative stress, metabolism); costly	Gold standard in nAMD and DR; evolving delivery systems for durability
Gene therapy	AAV-mediated expression of anti-angiogenic or neuroprotective proteins	Long-term expression; potential single-dose treatment; targets disease origin	Technical complexity; immune risks; uncertain durability; high cost	Early clinical trials show promising sustained VEGF suppression
Cell therapy (RPE/retinal progenitors)	Replacement of damaged retinal or RPE cells; trophic factor release	Restores structure and function; potential regenerative effect	Surgical risks; graft rejection; integration challenges	Ongoing early-phase trials in AMD
Nutraceuticals/dietary supplements	Antioxidant, anti-inflammatory, mitochondrial support	Noninvasive; suitable for early intervention; generally safe	Variable composition; limited standardization and clinical validation; dependent on early diagnosis	Adjunctive or preventive role; supported by preclinical evidence and selective clinical data
AI-assisted/precision medicine integration	Imaging, genetic, and metabolic data modeling for individualized care	Enables early detection, risk stratification, and optimized therapy	Requires robust data infrastructure and regulatory adaptation	Rapidly evolving; expected to enhance personalization of treatment

The table highlights complementary strengths and limitations of established pharmacologic interventions and emerging modalities, emphasizing that integrated, personalized care—combining pharmacologic, genetic, cellular, and nutraceutical approaches—offers the most promising path toward durable vision preservation.

On the far horizon, whole-eye transplantation (WET) remains a formidable but increasingly plausible goal. While immense challenges in optic nerve regeneration, immunocompatibility, and surgical revascularization persist, coordinated research initiatives are making incremental progress in *ex vivo* organ preservation and microsurgical techniques (Scarabosio et al., 2024; Advanced Research Projects Agency for Health (ARPA-H), 2024). The scientific and clinical knowledge generated by the pursuit of WET is, in itself, valuable, likely yielding spin-off technologies that will benefit the broader fields of neuroprotection and ocular repair.

## Toward an integrated and personalized management paradigm

The future of retinal care will not be defined by a single technology but by the strategic integration of these advances into a cohesive, patient-centric framework. The vision is a predictive, preventive, and personalized model that moves beyond reactive intervention.

In this integrated model, AI will function as the central nervous system of clinical decision-making. It will facilitate early detection through accessible screening, stratify patients based on their genetic and phenotypic risk profile, and provide dynamic, data-driven recommendations for management. This intelligence will guide the deployment of long-acting therapeutics, such as gene therapy, for patients identified as needing sustained VEGF suppression, while reserving conventional agents for others or for acute exacerbations.

This synergy extends to combining modalities; for example, patients receiving RPE cell transplants may be co-managed with tailored pharmacotherapy or gene-based neuroprotective strategies to support graft integration and function. The overarching objective is to create a layered defense: leveraging AI and lifestyle/nutraceutical strategies for primary prevention in high-risk individuals, employing durable biologics and gene therapies for secondary prevention and long-term control, and utilizing advanced surgical and regenerative techniques for structural restoration in advanced disease.

Nonetheless, the integration of nutraceuticals within personalized ophthalmic care also requires careful consideration of safety and potential risks. Although generally regarded as safe, unsupervised or excessive use of certain supplements—particularly fat-soluble vitamins, minerals, or concentrated botanical extracts—can lead to metabolic imbalance or toxicity, especially in elderly or poly-medicated patients. Moreover, concurrent administration with pharmacological therapies may result in unforeseen pharmacodynamic or pharmacokinetic interactions that could compromise treatment efficacy or amplify side effects (Weaver and Miller, 2017; Coates et al., 2024; Geller et al., 2015; Lawrenson and Grzybowski, 2015; Mirmiran et al., 2021). These concerns reinforce the need for standardized formulations, evidence-based dosing, and medical supervision when nutraceuticals are incorporated into integrative care models. Within a truly personalized framework, AI-driven risk stratification and biomarker monitoring could further mitigate these hazards by guiding appropriate combinations and dosing schedules (Massimino et al., 2023; Oliver Olid et al., 2024).

**Figure 5:**
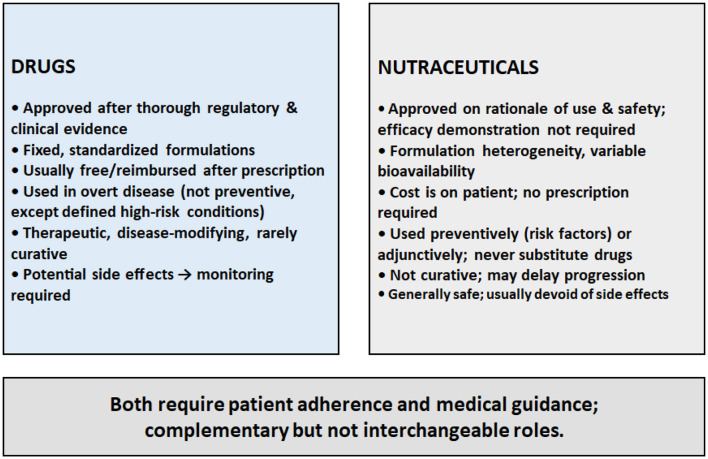
Summary: Drugs vs. nutraceuticals in retinal diseases. The scheme contrasts the regulatory and clinical profiles of drugs vs. nutraceuticals. Drugs and nutraceuticals differ in regulation, formulation, and clinical use: drugs are evidence-based therapies for overt disease, while nutraceuticals are safe adjuncts or preventive tools, never substitutes.

Therefore, ophthalmic research is building a future where the management of retinal diseases is less burdensome, more effective, and initiated earlier in the disease course. In summary, the path forward for retinal medicine lies in the creation of a predictive, personalized, and participatory model of care. By merging the strengths of AI, gene and cell therapy, pharmacology, and regenerative surgery, the field can move from reactive treatment of advanced pathology toward proactive and integrated preservation of vision (Figure 5).

## Conclusions

The clinical utility of nutraceuticals in retinal disease remains scientifically intriguing but clinically unresolved. Most of the current evidence arises from preclinical studies demonstrating antioxidant, anti-inflammatory, and antiangiogenic effects in animal or cellular models. Yet, doses effective in experimental settings are often far higher than those achievable or safe in humans, limiting direct translation. Human studies, when available, yield mixed results, constrained by heterogeneous formulations, inconsistent dosing, and limited trial quality.

For practicing ophthalmologists, the standard of care remains firmly pharmacologic—especially anti-VEGF therapies—whose efficacy is supported by large randomized trials and established guidelines. By contrast, nutraceutical use is largely empirical: although the AREDS and AREDS2 formulations provided proof-of-concept, most marketed supplements lack robust, reproducible clinical validation. Regulatory variability, coupled with inconsistent composition and marketing claims, further complicates interpretation and clinical adoption. Real-world effectiveness of nutraceuticals is inherently difficult to quantify and likely depends on early initiation, adherence, and individual patient factors such as genetic background, diet, and comorbidities. Consequently, their potential role should be viewed within a personalized, preventive framework rather than as a substitute for established pharmacotherapy. In sight-threatening conditions such as neovascular AMD or proliferative diabetic retinopathy, anti-VEGF drugs remain indispensable. In earlier or high-risk stages, selected, well-characterized nutraceutical formulations may confer modest adjunctive benefit, provided they are used judiciously and in conjunction with clinical monitoring.

Thus, the most balanced stance is neither blanket endorsement nor outright rejection. Instead, ophthalmologists should treat nutraceuticals as potential adjuncts, especially in preventive or early contexts. Early detection is crucial if we hope nutraceuticals to play a useful role in slowing progression of proliferative retinopathies. In any case, clinicians should insist on product quality, thoughtful patient selection, and ongoing reassessment as evidence evolves. Until more definitive data emerge, it is prudent to prioritize pharmacologic therapies and to counsel patients that long-term supplement regimens entail uncertain benefits and costs.

Looking ahead, meaningful integration of nutraceuticals into the future of retinal care—a future shaped by artificial intelligence and personalized medicine—will require progress on multiple fronts: regulatory reform, biomarker-guided patient stratification, rigorous large-scale trials, and a cultural shift within ophthalmology toward preventive strategies. Until that time, the most responsible path forward combines informed skepticism with open inquiry, always anchored in a commitment to evidence-based, patient-centered care.
